# Optimization of Pickling Solution for Improving the Phosphatability of Advanced High-Strength Steels

**DOI:** 10.3390/ma14010233

**Published:** 2021-01-05

**Authors:** Sangwon Cho, Sang-Jin Ko, Jin-Seok Yoo, Joong-Chul Park, Yun-Ha Yoo, Jung-Gu Kim

**Affiliations:** 1 Department of Materials Science and Engineering, Sungkyunkwan University, Suwon-Si 16419, Korea; ppkh@skku.edu (S.C.); tkdwls121@skku.edu (S.-J.K.); wlstjr5619@skku.edu (J.-S.Y.); 2 Steel Solution Marketing Dept., POSCO Global R&D Center, Incheon 21985, Korea; heavymetal@posco.com (J.-C.P.); yunha778@posco.com (Y.-H.Y.)

**Keywords:** advanced high-strength steel, phosphate coating, acid cleaning, electrochemical impedance spectroscopy

## Abstract

This study investigated the optimum pickling conditions for improving the phosphatability of advanced high-strength steel (AHSS) using surface analysis and electrochemical measurements. To remove the SiO_2_ that forms on the surface of AHSS, 30 wt.% NH_4_HF_2_ was added to the pickling solution, resulting in a significant reduction in the amount of SiO_2_ remaining on the surface of the AHSS. The phosphatability was improved remarkably using HNO_3_ concentrations higher than 13% in the pickling solution. Furthermore, phosphate crystals became finer after pickling with a HNO_3_-based solution rather than a HCl-based solution. Electrochemical impedance spectroscopy (EIS) data indicated that the corrosion resistance of AHSS subjected to HNO_3_-based pickling was higher than that of AHSS subjected to HCl-based pickling. Fluorine compounds, which were involved in the phosphate treatment process, were only formed on the surface of steel in HNO_3_-based solutions. The F compounds reacted with the phosphate solution to increase the pH of the bulk solution, which greatly improved the phosphatability. The phosphatability was better under HNO_3_-based conditions than a HCl-based condition due to the fineness of the phosphate structure and the increased surface roughness.

## 1. Introduction

Consumer demand for automobiles has been increasing over the last decade, with particular regard for features such as appearance, driving performance, comfort, safety, fuel efficiency, and environmental friendliness. In response, the automotive industry has spurred the development of new and advanced technologies to improve safety while reducing vehicle weight. However, the technology used to manufacture lightweight automobiles is associated with environmental problems [1]. Research into automotive weight reduction can be divided into the fields of lightweight materials and high-strength steel sheets [2]. Studies on the use of lightweight materials for automobiles have mainly focused on aluminum and fiber-reinforced plastics [3,4,5], but the practical use of these materials is difficult due to product design limitations and manufacturing problems. However, high-strength materials can be used without making significant changes to conventional manufacturing methods, so they have the advantage of minimal additional investment costs. In addition, multi-material applications can be used with high-strength materials, depending on the function of the component; thus, a variety of designs are possible. Another advantage of using high-strength materials is that reductions in the thickness and weight of the material are obtained at the same time. Therefore, various grades of steel, from mild steel to advanced high-strength steel (AHSS), have been used in recent automotive bodies. In addition, steel manufacturers are actively researching applications for more advanced steel sheets to further reduce the weight.

Generally, a steel sheet for automobiles is subjected to a phosphate treatment to improve corrosion resistance and ensure coating film adhesion before painting [6,7]. Phosphate crystals form on the steel sheet surface from the phosphate treatment, which greatly enhances the adhesion of electro-painting [7]. The size of the phosphate crystals and coating weight are very important factors: relatively small crystals result in the formation of denser phosphate crystals, resulting in better adhesion to the coating.

During phosphate treatment, an acidic zinc phosphate solution reacts with the surface of the metal and consumes hydrogen ions in the solution. This reaction increases the pH near the metal surface. Then, the phosphate solution is saturated with zinc phosphate. As a result of the increase in pH, the formation of phosphate crystals is promoted. Therefore, phosphatability can be improved by an accelerator that increases the rate of hydrogen ion consumption on the metal surface [8].

Alloy elements such as Si and Mn are indispensable elements for obtaining high strength and high ductility. Therefore, AHSS has a higher content of these alloying elements compared to general steels. However, during steel manufacturing and use, Si and Mn oxides are easily formed on the steel surface. When these composite oxides densely form on the surface of a steel sheet, they have a significant effect on the phosphate treatment. In particular, a Si oxide film that forms on the AHSS surface acts as a barrier to phosphating, thereby decreasing the phosphatability [9]. Therefore, to improve the phosphatability of AHSSs that contain a large amount of Si, the formation of these oxides on the surface of the steel must be suppressed.

Currently, two main methods are used to remove the Si oxide that forms on the surface of AHSS. The first method uses a high-temperature, high-concentration inorganic acid (e.g., hydrochloric acid, sulfuric acid, nitric acid, or formic acid) solution, which removes the Si oxide by dissolving the base metal under the Si oxide [10]. In this method, if the concentration of the inorganic acid is low, the Si oxide on the surface is not removed; conversely, if the concentration is too high, over-etching occurs and the material cannot be used in automotive manufacturing. Therefore, it is important to appropriately control the concentration of the inorganic acid.

The second method utilizes hydrofluoric acid (HF) in a pickling solution to remove the Si oxide, using the following reaction:

SiO_2_ + 4HF → SiF_4_ + 2H_2_O (1)

This method does not use HF alone; rather, HF is mixed with an inorganic acid to activate its reaction [11]. However, because HF is very dangerous, it is challenging to use it in a pickling solution. To react with silicate, which is a basic form of Si oxide, F^−^ is required. Therefore, many researchers have attempted to replace HF with ammonium fluoride (NH_4_F) and ammonium hydrogen fluoride (NH_4_HF_2_) as a new source of F^–^ because they are eco-friendly and less hazardous than HF. NH_4_F and NH_4_HF_2_ have been used in a mixture with an inorganic acid solution; the etching rate can be adjusted by controlling the type and concentration of the inorganic acid [12]. Because NH_4_HF_2_ has more F than NH_4_F, its etching efficiency is better and its cost is lower; thus, it is suitable as a HF substitute.

**Figure 1:**
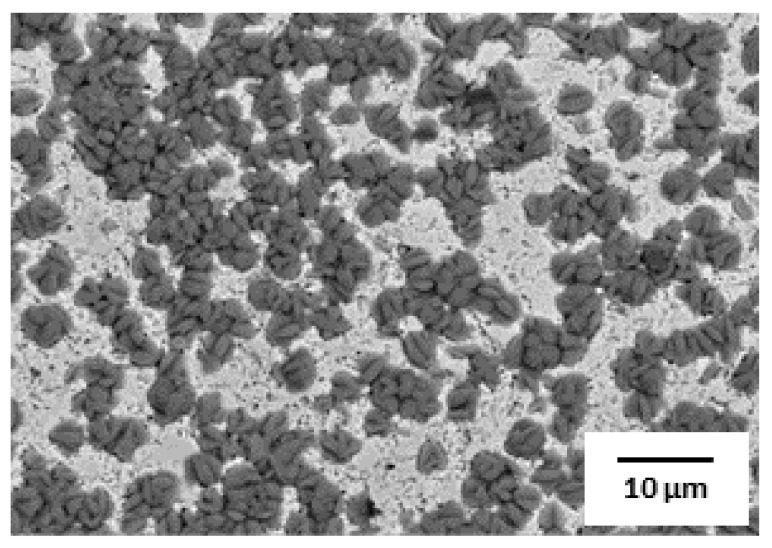
Surface SEM image of advanced high-strength steel (AHSS) after phosphate treatment under the conventional pickling condition.

Although many studies on the phosphatability of AHSS have been carried out, some problems still exist, such as a decrease in phosphatability due to Si oxides. The phosphatability of AHSS under the conventional pickling conditions used in industry are not optimal; as shown in Figure 1, the coating coverage and the coating weight are insufficient. Therefore, in this study, we investigated the optimum pickling conditions to improve the phosphatability of AHSS while minimizing environmental hazard.

## 2. Materials and Methods

The specimens used in the experiment were AHSSs with Si contents of 0.4 and 1.0, as described in Table 1. The specimens were dipped in an alkaline solution at 45 °C for 2 min for degreasing before testing. The phosphate treatment process was divided into two steps: pickling and phosphating. As listed in Table 2, the pickling solutions used in the experiment were prepared by adding 30 wt.% NH_4_HF_2_ to HCl and HNO_3_ at various concentrations (5.5, 8, 10.5, 13, 15.5, and 18 wt.%). Pickling tests were performed at 55 °C for 7 s. Then, after a degreasing and surface conditioning process, phosphate treatment was performed in a Zn–phosphate solution according to the common automotive process.

Phosphatability was evaluated by coating coverage and coating weight. In an image obtained using the back scattered electron (BSE) mode in scanning electron microscopy (SEM; JSM-6700F, JEOL Ltd., Akishima, Japan), a difference in brightness existed between the areas where phosphate crystals were formed and where they did not form. The difference between the light and dark was measured to determine coating coverage using Image J S/W (Laboratory for Optical and Computational Instrumentation, University of Wisconsin). The coating weight was analyzed using energy dispersive X-ray fluorescence (EDXRF; EDX-8000, SHIMADZU, Kyoto, Japan). After calibrating the P signal detected in a standard specimen of known phosphate coating thickness, the coating weight can be calculated by multiplying the measured P signal by density. The oxides that remained on the steel surface after pickling were analyzed using secondary ion mass spectrometry (SIMS; TOF-SIMS-5, Ion-ToF company, Munster, Germany), X-ray diffraction (XRD; D/max-2500V/PC, Rigaku, Tokyo, Japan), energy dispersive spectroscopy (EDS; JEOL JSM-6700F, JEOL Ltd., Akishima, Japan), and an electron probe micro-analyzer (EPMA; JEOL, JXA-8530F, JEOL Ltd., Akishima, Japan).

Electrochemical impedance spectroscopy (EIS; VSP300, Neoscience, Seoul, Korea) tests were performed with an amplitude of 10 mV in the frequency range of 100 kHz to 1 mHz. The electrochemical cell consisted of a three-electrode system. The counter electrode was a graphite rod, and a saturated calomel electrode was used as the reference electrode. The area exposed to the electrolyte was 1 cm^2^. The test solution was a cyclic corrosion test solution from the Society of Automotive Engineering (SAE solution), and its chemical composition is listed in Table 3. EIS data were fit to the form of a Bode plot, and Bode spectra were fit to equivalent circuit models by using ZSimpWin software (Ver. 3.21).

To analyze the pH change of the phosphate solution due to the oxides, 1 M of the iron oxides and the F compounds generated during pickling were dissolved in the phosphate solution. The change in pH was measured using a pH meter. To analyze the correlation between surface roughness and phosphatability, the surface roughness of the specimen after pickling was measured using an α-step (Alpha-Step IQ, KLA-Tencor, Milpitas, CA, USA).

## 3. Results and Discussion

### 3.1. Surface Analysis

**Figure 2:**
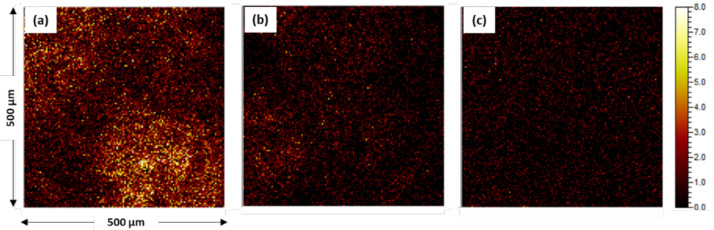
TOF-secondary ion mass spectrometry (SIMS) images of SiO_2_ remaining on the 1.0Si steel surface after pickling under (**a**) conventional condition, (**b**) a HCl-based condition, and (**c**) a HNO_3_-based condition.

**Figure 3:**
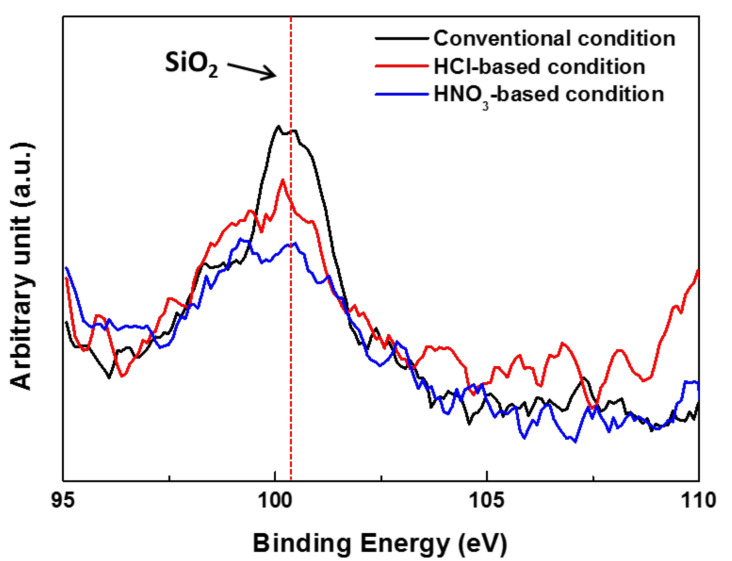
XPS spectra of Si on the 1.0Si steel surface after pickling.

Figure 2 and Figure 3 show the amount of SiO_2_ remaining on the 1.0Si steel surface layer as analyzed by TOF-SIMS and XPS after pickling with various solutions. Figure 2a shows a conventional pickling condition, in which the amount of SiO_2_ remaining on the surface is larger and the distribution of SiO_2_ is more uneven than under the other pickling conditions. In Figure 2b,c, however, in the pickling solutions with added NH_4_HF_2_, the SiO_2_ on the steel surface was noticeably reduced, even though it was not completely removed. Figure 3 shows that the intensity of the SiO_2_ peak in the pickling solution with added NH_4_HF_2_ was reduced compared with the intensity of the SiO_2_ peak under conventional pickling conditions. Thus, the amount of SiO_2_ remaining on the surface was significantly reduced by NH_4_HF_2_-added pickling.

**Figure 4:**
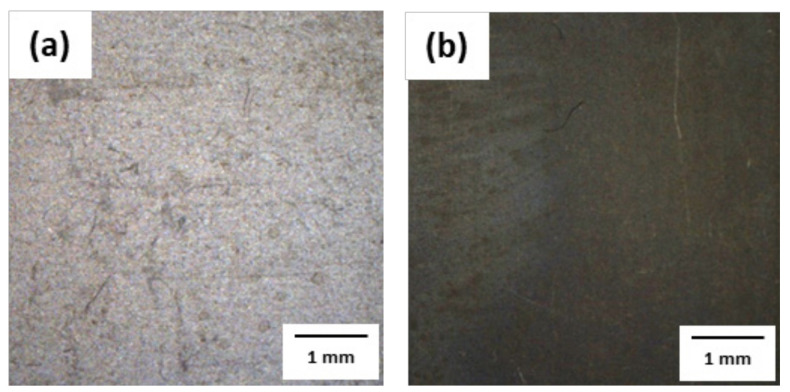
Optical microscopy (OM) images of the 1.0Si steel surface after pickling under (**a**) a HCl-based condition and (**b**) a HNO_3_-based condition.

**Figure 5:**
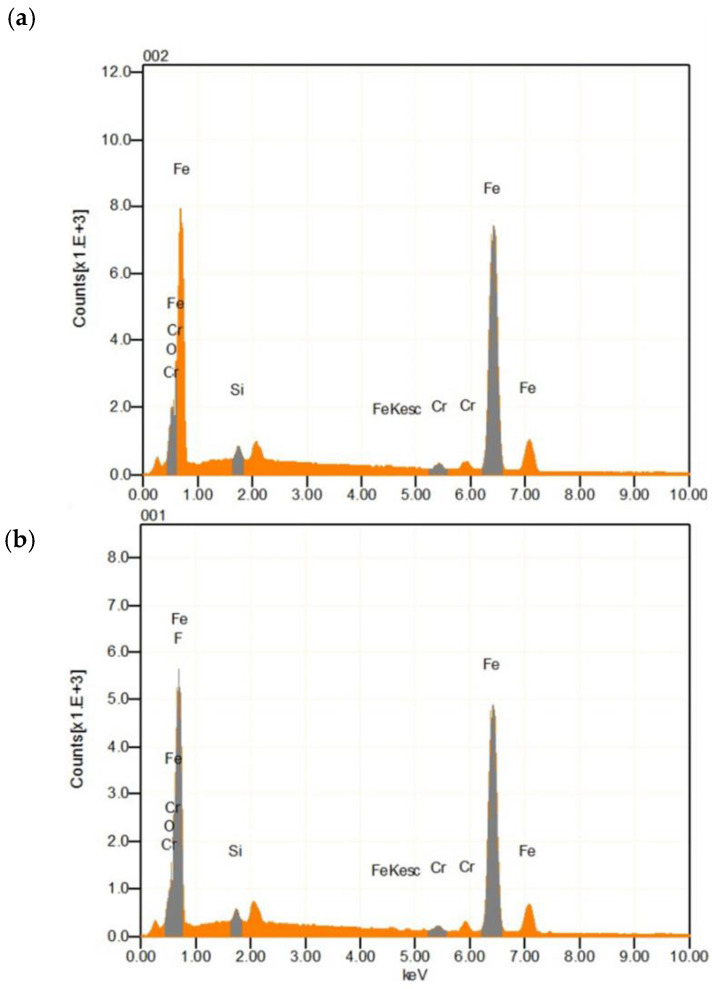
EDS analysis of the 1.0Si steel surface after pickling under (**a**) a HCl-based condition and (**b**) a HNO_3_-based condition.

**Figure 6:**
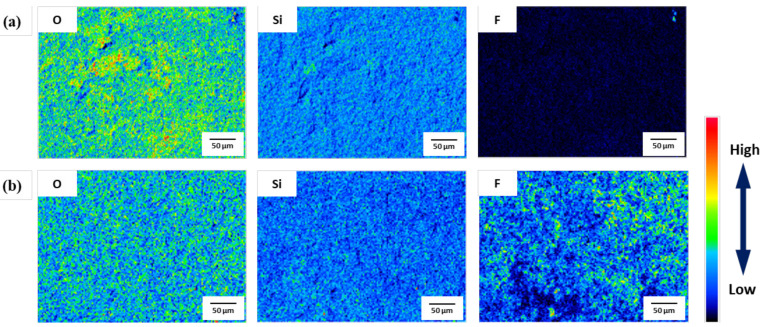
EPMA analysis of the 1.0Si steel surface after pickling under (**a**) a HCl-based condition and (**b**) a HNO_3_-based condition.

**Figure 7:**
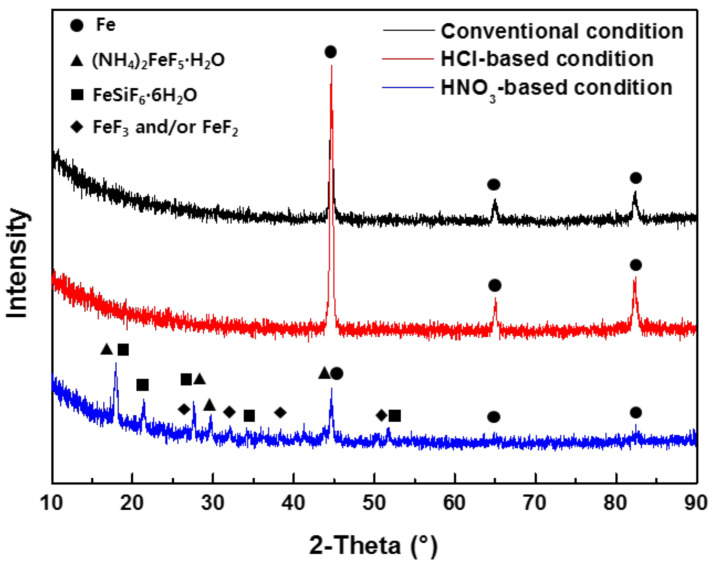
XRD analysis of the 1.0Si steel surface after pickling.

After the pickling of 1.0Si steel under various conditions, the surface was analyzed with optical microscopy (OM), as shown in Figure 4. The surface of the specimen before and after HCl-based pickling was the same; however, after HNO_3_-based pickling, the surface of the specimen was uniformly covered with a new gray product. Figure 5, Figure 6 and Figure 7 and Table 4 reveal the oxides that remained on the surface after pickling, as analyzed by EDS, EPMA, and XRD.

As shown in Figure 5, the EDS peaks of O, Si, Cr, and Fe can be observed from 1.0Si steel under all pickling conditions, indicating that the remaining oxides on the surface were mainly Fe, Cr, and Si oxides. However, the F peak was observed only under HNO_3_-based pickling conditions. The results of EPMA analysis (Figure 6) were similar to those of EDS: an F component was observed throughout the surface only under HNO_3_-based pickling conditions. This was largely due to NH_4_HF_2_, which means that the F compounds were formed on the surface only after HNO_3_-based pickling. XRD analysis (Figure 7) indicated that the Fe of the base material was only detected in the specimens that underwent conventional pickling and HCl-based pickling. However, various components were detected in the specimen that underwent HNO_3_-based pickling. The products formed under HNO_3_-based pickling were (NH_4_)FeF_5_∙H_2_O, FeSiF_6_∙6H_2_O, FeF_2_, and/or FeF_3_. The products were produced by the following reactions [13,14]:

Fe_2_O_3_ + 5NH_4_HF_2_ → 2(NH_4_)_2_FeF_5_ + 3H_2_O + NH_3_↑ (2)

Si + 2H^+^ + 2F^-^ + 4HF → H_2_SiF_6_∙6H_2_O + 2H_2_↑ (3)

Fe^2+^ + 2F^-^ → FeF_2_ (4)

FeF_2_ + H_2_SiF_6_ → FeSiF_6_ + 2HF (5)

In the HNO_3_/HF system, FeF_3_·3H_2_O was in a stable phase at low pH (pH < 3.16) and then transformed into FeF_2_ and FeF_3_ at higher pH [15,16]. However, in the HCl/HF system, HCl made insoluble ferric fluoride, preventing the formation of fluoride precipitates [17].

### 3.2. Phosphatability

**Figure 8:**
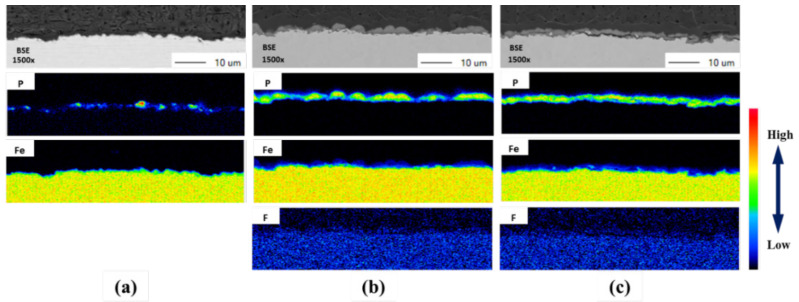
EPMA images of cross sections of 1.0Si steel formed with phosphate coating using (**a**) conventional conditions, (**b**) a HCl-based condition, and (**c**) a HNO_3_-based condition.

Figure 8 shows EPMA images of a cross section of 1.0Si steel on which a phosphate coating was formed. Under conventional pickling conditions, phosphate crystals were rarely formed; under HCl-based pickling, the areas where phosphate crystals did not form were sparse. However, under HNO_3_-based pickling, the phosphate crystals were uniformly formed. As shown in Figure 5 and Figure 6, the F compounds found after pickling were not observed after the phosphate treatment. Therefore, the F compounds were involved in the formation of phosphate crystals. In other words, the F compounds introduced by pickling had a positive effect on the phosphatability of the steel.

**Figure 9:**
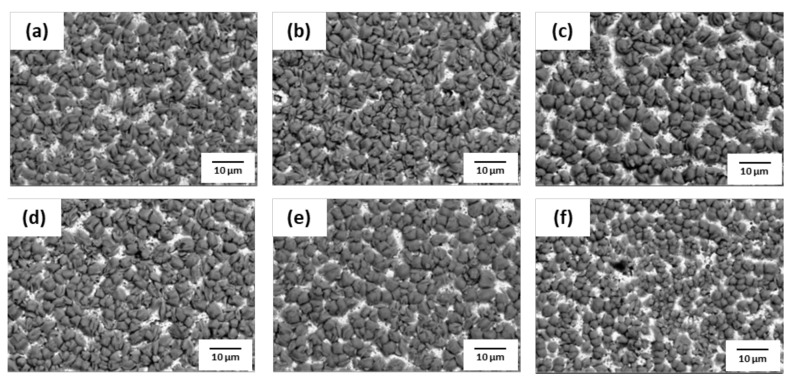
Surface SEM images of 1.0Si steel after phosphate treatment under HCl-based pickling condition with HCl concentration of (**a**) 5.5%, (**b**) 8%, (**c**) 10.5%, (**d**) 13%, (**e**) 15.5%, and (**f**) 18%.

**Figure 10:**
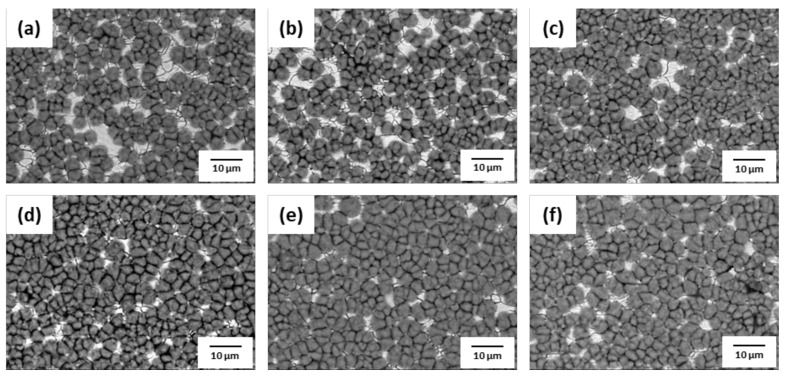
Surface SEM image of 0.4Si steel after phosphate treatment under HCl-based pickling condition with HCl concentration of (**a**) 5.5%, (**b**) 8%, (**c**) 10.5%, (**d**) 13%, (**e**) 15.5%, and (**f**) 18%.

**Figure 11:**
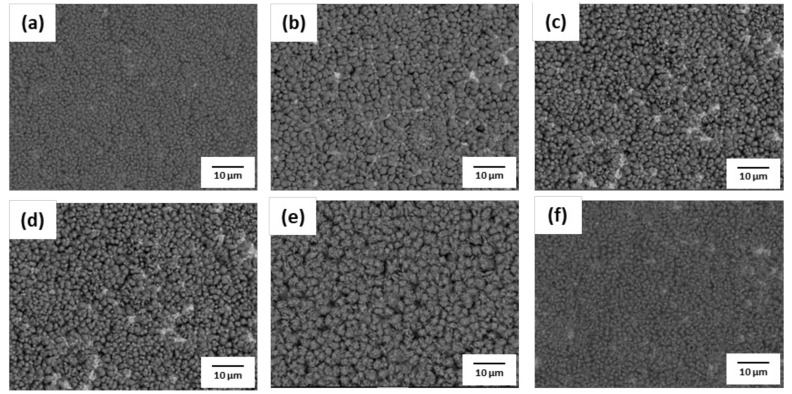
Surface SEM images of 1.0Si steel after phosphate treatment under HNO_3_-based pickling condition with HNO_3_ concentration of (**a**) 5.5%, (**b**) 8%, (**c**) 10.5%, (**d**) 13%, (**e**) 15.5%, and (**f**) 18%.

**Figure 12:**
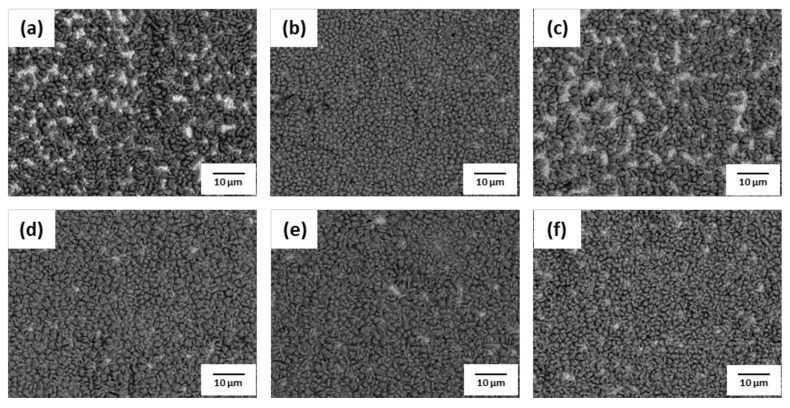
Surface SEM images of 0.4Si steel after phosphate treatment under HNO_3_-based pickling condition with HNO_3_ concentration of (**a**) 5.5%, (**b**) 8%, (**c**) 10.5%, (**d**) 13%, (**e**) 15.5%, and (**f**) 18%.

The phosphate treatment of AHSS was performed with various concentrations of hydrochloric acid (HCl) and nitric acid (HNO_3_). SEM images of the surface after phosphate treatment are shown in Figure 9, Figure 10, Figure 11 and Figure 12. When pickling was performed with NH_4_HF_2_, more phosphate crystals were formed compared to with the conventional pickling. However, as shown in Figure 9 and Figure 10, phosphate crystals were not formed in many regions. Figure 11 and Figure 12 show significantly fewer areas where phosphate crystals were not formed compared to Figure 9 and Figure 10. Furthermore, the phosphate crystals became finer after pickling with a HNO_3_-based solution than with a HCl-based solution. In addition, phosphate crystals were formed more uniformly on the surface of 1.0Si steel in both the HCl- and HNO_3_-based solutions compared to those formed on 0.4Si steel.

**Figure 13:**
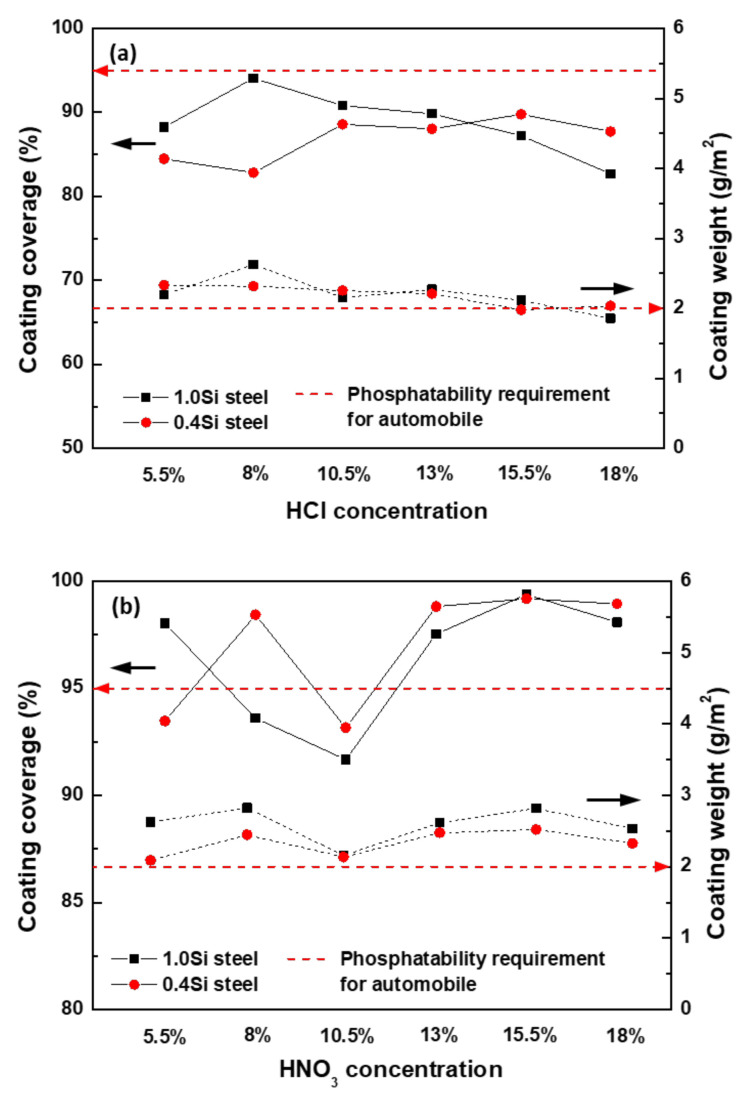
Coating coverage and coating weight vs. the inorganic acid concentration under (**a**) HCl-based condition and (**b**) HNO_3_-based condition.

Figure 13 shows the coating coverage and coating weight as a function of the HCl and HNO_3_ concentrations. Figure 13a shows that the coating coverage of 1.0Si steel decreased as the concentration of hydrochloric acid increased, whereas the coating coverage of 0.4Si steel increased as the concentration of hydrochloric acid increased. In addition, the coating coverage of both steels did not meet the phosphate quality requirements (95% or more) for automobiles. In the case of the coating weight, as the concentration of HCl increased, the coating weight of both 1.0Si and 0.4Si steel decreased. However, the phosphate quality requirement (2 g/m^2^ or more) for automobiles was satisfied at all HCl concentrations except for 18%. As shown in Figure 13b, the coating coverage was not uniform at HNO_3_ concentration below 10.5%, whereas HNO_3_ concentration of 13% or more showed excellent coating coverage of at least 98%. A HNO_3_ concentration of 8% was sufficient to achieve the desired coating coverage for 0.4Si steel. However, 1.0Si steel does not satisfy the desired coating coverage. Therefore, at least 13% of HNO_3_ was required to meet the desired coating coverage. Furthermore, the coating weight was excellent at all concentrations.

### 3.3. Electrochemical Imedance Spectroscopy

**Figure 14:**
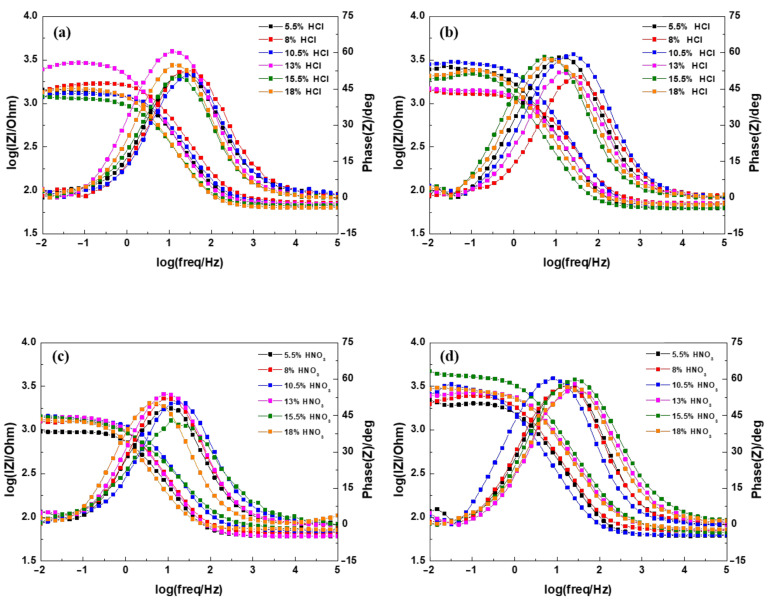
Bode impedance plots of electrochemical impedance spectroscopy (EIS) data of (**a**,**c**) 1.0Si steel and (**b**,**d**) 0.4Si steel in SAE solution.

The results of EIS in the form of a Bode plot are shown in Figure 14. In the Bode plot, the low-frequency region is related to the surface film and the metal/surface interface, whereas the high-frequency region is attributed to the defects on the metal surface [18,19,20]. In the low-frequency region, the impedance (|Z|) values of 0.4Si steel were higher than those of 1.0Si steel. However, there was no significant difference in |Z| value with the use of different types of inorganic acids in both steels. The phase angle maxima and shoulder widths of the phase angles also showed the same trends as the |Z| values. The results of |Z| values and phase angle data indicate that the corrosion resistance of 0.4Si steel was superior to that of 1.0Si steel. However, there was no relationship between the concentration of inorganic acid and the |Z| values. Furthermore, improvement in corrosion resistance from the phosphate coating was not significant due to the short phosphating time used in the experiment. In general, the phosphate coating improves the corrosion resistance of a substrate. However, noticeable improvement in corrosion resistance is not observed during the stage of induction and commencement of film growth, which are the stages from the start of the phosphate treatment to 3 min [21,22]. Since the phosphating time in this study was within 2 min, the EIS results indicate that phosphate crystals had not sufficiently formed to be able to improve corrosion resistance for both types of inorganic acid. The phosphating industry generally uses coating weight and coverage as part of quality control, but coating weight and coverage do not have a direct relationship to corrosion resistance [21]. Therefore, the low correlation between coating weight, coverage, and the |Z| values was due to various factors, such as thickness and structure homogeneity.

**Figure 15:**
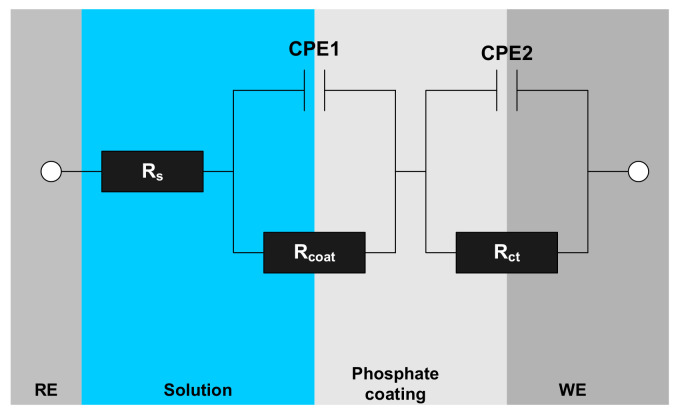
Equivalent circuit for phosphate coated steel in SAE solution. R_s_ is the solution resistance; CPE1 and R_coat_ are the capacitance and resistance of the phosphate coating layer; CPE2 is the double layer capacitance; R_ct_ is the charge transfer resistance; WE and RE are working electrode and reference electrode.

**Figure 16:**
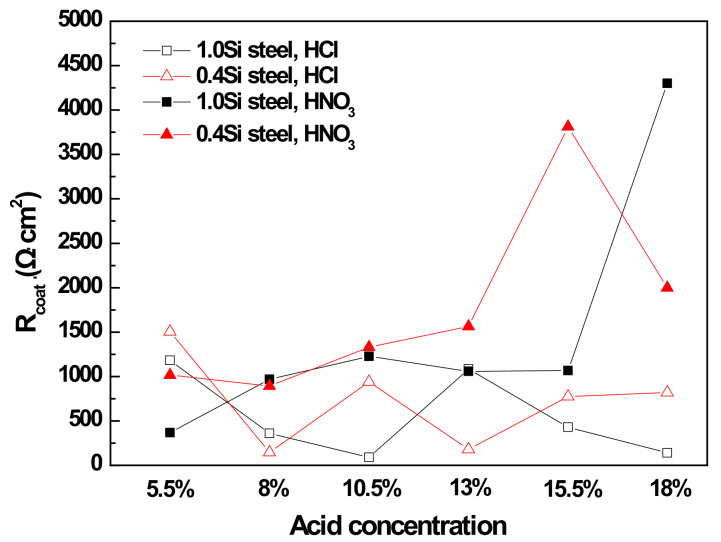
Variation of coating resistance (R_coat_) from EIS analysis vs. inorganic acid concentration.

The equivalent electric circuit model used to determine the optimized value for resistance and capacitance parameters is shown in Figure 15, and the corresponding EIS results are listed in Table 5. R_s_ is the solution resistance, CPE1 and R_coat_ are the capacitance and resistance of the phosphate coating layer, CPE2 is the double layer capacitance, R_ct_ is the charge transfer resistance, Q_coat_ and Q_ct_ are coating’s capacitance and charge transfer capacitance, and n is an empirical exponent (0 ≤ n ≤ 1) measuring the deviation from the behavior of the ideal electric capacity. Figure 16 shows the variation of coating resistance (R_coat_) from EIS measurement results as a function of inorganic acid concentration.

Unlike the |Z| values, there was no significant difference between the R_coat_ of 1.0Si and 0.4Si steel. However, the R_coat_ under HNO_3_-based pickling conditions was higher than that under HCl-based pickling condition in both steels, and R_coat_ increased significantly as the concentration increased under HNO_3_-based pickling conditions. This result is consistent with the coating coverage data shown in Figure 6. R_coat_ was affected more by coating coverage than by coating weight.

### 3.4. pH Measurement

**Figure 17:**
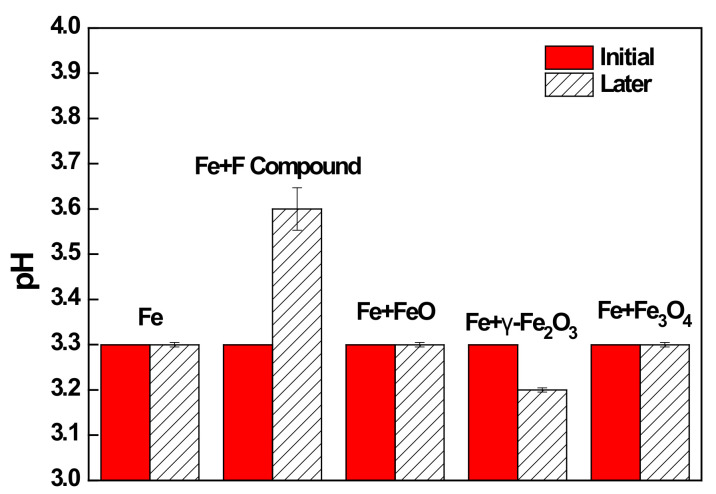
pH variation of phosphate solution before and after the addition of various iron oxides and F compounds.

In general, during the early stages of phosphate treatment, the reaction of iron or iron oxide with hydrogen ions increases locally with the pH at the metal surface, which promotes the nucleation of phosphate crystals [8]. The effects of iron oxides and F compounds generated under HNO_3_-based pickling on the pH of the phosphate solution, are shown in Figure 17. The pH of the bulk solution did not change significantly when iron and iron oxides reacted with the phosphate solution. However, with F compounds, the pH of the bulk solution increased from 3.3 to 3.6 after reacting with the phosphate solution. Among F compounds, FeF_3_ has a pH of 3.5–4.0 when dissolved in water (Equation (6)) [23]. Therefore, this F compound could have increased the pH of the phosphate solution by reacting with water. This increase in the pH of the phosphate solution indicates that the F compounds had a positive effect on the phosphatability of the steel because F compounds act as an accelerator for consumption of hydrogen ions.

FeF_3_ + 3H_2_O → Fe(OH)_3_ + 3HF (6)

### 3.5. Surface Roughness Measurement

**Figure 18:**
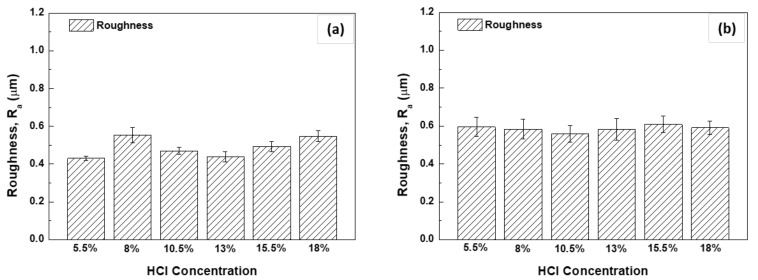

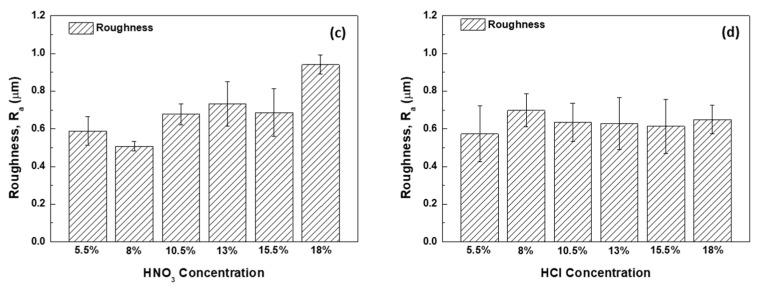
Correlation between surface roughness and phosphatability; (**a**,**c**) 1.0Si steel and (**b**,**d**) 0.4Si steel.

The correlation between the surface roughness after pickling and the phosphatability of the steel is shown in Figure 18. The surface roughness was higher under a HNO_3_-based pickling condition than under a HCl-based pickling condition. This result is due to NO_3_^−^ ions acting as oxidizing agents; thus, the corrosiveness of HNO_3_ is higher than that of HCl [24]. Generally, a higher surface roughness is associated with a higher coating weight and the formation of finer crystals; a level of 0.76–1.77 μm was reported to be the most suitable average roughness (Ra) [21,25]. In other words, the phosphatability was better with HNO_3_-based pickling than with HCl-based pickling due to the surface roughness. However, the phosphatability did not have a significant correlation with surface roughness.

## 4. Conclusions

In this study, the optimum pickling conditions to improve the phosphatability of AHSS were investigated using various types of surface analysis and EIS. The conclusions based on our investigation are as follows:

- With HNO_3_-based pickling solutions, the phosphatability improved remarkably at HNO_3_ concentrations higher than 13% for both steels. Furthermore, the phosphate crystals became finer after pickling with a HNO_3_-based solution compared to those with a HCl-based solution.
- SiO_2_ was noticeably removed by a pickling solution with NH_4_HF_2_.
- The corrosion resistance of phosphate-treated AHSS was higher using a HNO_3_-based pickling condition compared to a HCl-based pickling condition.
- With HNO_3_-based pickling solutions, F compounds, which are involved in the phosphate treatment process, formed on the surface of the AHSS. The F compounds reacted with the phosphate solution to increase the pH of the phosphate solution, thereby greatly improving the phosphatability of AHSS.
- The phosphatability was better under HNO_3_-based pickling conditions than under HCl-based pickling conditions due to the increased surface roughness.

## Figures and Tables

**Table 1:** Chemical compositions of the steels. (Unit: wt.%).

Grade	C	Mn	Si	P	S
**0.4Si steel**	0.17	2.8	0.25	0.02	0.005
**1.0Si steel**	0.1	2.8	1.2	0.03	0.003

**Table 2:** Pickling conditions for the steels.

Parameter	Conventional Pickling Condition	HCl-Based Pickling Condition	HNO_3_-Based Pickling Condition
**Inorganic acid**	HCl	HCl	HNO_3_
**Acid concentration (wt.%)**	5.5, 18	5.5, 8, 10.5, 13, 15.5, 18	5.5, 8, 10.5, 13, 15.5, 18
**Additives**	−	NH_4_HF_2_ (30 wt.%)	NH_4_HF_2_ (30 wt.%)
**Temperature (°C)**	55	55	55
**Time (s)**	Within 7	7	7

**Table 3:** Chemical composition of Society of Automotive Engineering (SAE) solution.

Chemicals	NaCl	CaCl_2_	NaHCO_3_	(NH_4_)_2_SO_4_
SAE solution (wt.%)	0.05	0.1	0.075	0.35

**Table 4:** EDS results of the 1.0Si steel surface after pickling.

Parameter	Fe	O	Si	F	Cr
**HCl-based** **pickling condition (%)**	94.13	3.77	0.85	−	1.25
**HNO_3_-based** **pickling condition (%)**	92.25	1.98	0.85	3.56	1.36

**Table 5:** Parameters from electrochemical impedance spectroscopy measurements.

Acid	Steel	Concentration	R_s_ (Ω∙cm^2^)	CPE1		R_coat_ (Ω∙cm^2^)	CPE2		R_ct_ (Ω∙cm^2^)
				Q_coat_ (μF/cm^2^)	n_1_		Q_ct_ (μF/cm^2^)	n_2_	
**HCl**	**1.0Si**	5.5%	62.98	56	0.8	1183	45.5	0.8	279.8
		8%	72.35	149	0.7301	358.2	42.4	0.9004	1285
		10.5%	63.58	178.3	0.5432	89.6	46.6	0.9395	1223
		13%	70.29	189.3	1	1083	64.7	0.9535	1777
		15.5%	67.04	108	0.8995	430.8	243	0.7957	673.2
		18%	63.41	208	0.975	140.6	104	0.8421	1,287
	**0.4Si**	5.5%	62.37	75	0.8419	1504	265	1	918.9
		8%	70.87	8990	0.6406	145.9	49.1	0.8305	1168
		10.5%	63.16	207.9	0.9996	940.4	45.8	0.8414	1878
		13%	70.72	981	1	179.4	70.6	0.8408	1186
		15.5%	62.67	179	0.8284	775.1	220	1	1320
		18%	69.29	134	0.8182	819.5	153	1	1413
**HNO_3_**	**1.0Si**	5.5%	60.91	476	1	367.8	157	0.8468	528.2
		8%	66.97	120	0.8452	967.2	910	0.98	235.9
		10.5%	70.41	74.2	0.8295	1227	14,200	0.9539	138.1
		13%	61.01	186	0.8669	1059	250	0.8649	301.2
		15.5%	72.76	266	0.6565	1066	204	0.8387	272.2
		18%	73.47	300.1	0.8455	4300	397.7	0.9307	766.8
	**0.4Si**	5.5%	61.46	72.2	0.8527	1015	169	0.982	945.6
		8%	70.7	209	1	892.6	65	0.8414	1525
		10.5%	62.75	345	1	1332	108	0.8473	1680
		13%	72.12	48.7	0.9522	1564	66.2	0.7682	1036
		15.5%	65.98	29.4	0.8391	3812	2085	0.4284	690.9
		18%	73.74	38.9	0.8746	2000	426	0.635	932.9

## Data Availability

Data sharing is not applicable to this article.
